# PanForest: predicting genes in genomes using random forests

**DOI:** 10.1093/bioinformatics/btag005

**Published:** 2026-01-09

**Authors:** Alan J S Beavan, Maria Rosa Domingo-Sananes, James O McInerney

**Affiliations:** School of Biological Science, Faculty of Biology, Medicine & Health, University of Manchester, Manchester, M13 9PL, United Kingdom; School of Life Sciences, The University of Nottingham, Nottingham NG7 2UH, United Kingdom; School of Science and Technology, Nottingham Trent University, Nottingham NG1 4FQ, United Kingdom; Department of Evolution, Ecology and Behaviour, University of Liverpool, Liverpool L69 3BX, United Kingdom

## Abstract

**Motivation:**

The presence or absence of some genes in a genome can influence whether other genes are likely to be present or absent. Understanding these gene co-occurrence and avoidance patterns reveals fundamental principles of genome organization, with applications ranging from evolutionary reconstruction to rational design of synthetic genomes.

**Results:**

PanForest, presented here, uses random forest classifiers to predict the presence and absence of genes in genomes from the set of other genes present. Performance statistics output by PanForest reveal how predictable each gene’s presence or absence is, based on the presence or absence of other genes in the genome. Further, PanForest produces statistics indicating the importance of each gene in predicting the presence or absence of each other gene. The PanForest software can run serially or in parallel, thereby facilitating the analysis of pangenomes at Network of Life scale.

A pangenome of 12 741 accessory genes in 1000 *Escherichia coli* genomes was analysed in around 5 h using eight processors. To demonstrate PanForest’s utility, we present a case study and show that certain genes associated with resistance to antimicrobial drugs reliably predict the presence or absence of other genes associated with resistance to the same drug. Further, we highlight several associations between those genes and others not known to be associated with antimicrobial resistance (AMR), or associated with resistance to other drugs. We envisage PanForest’s use in studies from multiple disciplines concerning the dynamics of gene distributions in pangenomes ranging from biomedical science and synthetic biology to molecular ecology.

**Availability and implementation:**

The software if freely available with a full manual and can be found with at www.github.com/alanbeavan/PanForest DOI: https://doi.org/10.5281/zenodo.17865482.

## 1 Introduction

Bacterial genomes vary significantly in their gene content both within and between species (Tettelin *et al.* 2005, Treangen and Rocha 2011, Puigbò *et al.* 2014, Vos *et al.* 2015, McInerney *et al.* 2017). The pangenome encompasses all genetic variation across a set of organisms. Here, we focus specifically on patterns of gene presence and absence. Previous studies have shown that the presence or absence of a gene can influence the likelihood of the presence or absence of other genes (Beavan and McInerney 2022, Caudal *et al.* 2022, Rosconi *et al.* 2022). This tells us not only about the plasticity of bacterial genomes but also allows us to ask questions about how genes might work cooperatively, or in conflict with one another, or indeed whether their fitness effects are entirely orthogonal to one another and exert little or no influence.

Rising AMR in pathogenic bacteria presents a major clinical challenge rooted in evolutionary biology. Continued antibiotic use increases the frequency of resistance alleles, threatening our ability to treat infections without new therapeutic approaches (Ho *et al.* 2025). Accordingly, understanding which features of a pathogen make it resistant to antimicrobials is central to improving clinical outcomes. Biomedical studies have identified antimicrobial properties in thousands of genes in pathogen genomes (Alcock *et al.* 2023), yet substantial gaps remain in mapping the complete genetic architecture underlying resistance mechanisms (Zhang *et al.* 2022).

Here, we present PanForest, a software package designed to make statistical predictions of a gene’s presence or absence in a genome based on the presence or absence of the other genes in the genome. The context of gene presence and absence across a pangenome dataset provides the data for these predictions. We anticipate that genes differ in their genetic context requirements, with some depending on supportive genetic networks for persistence, while others are prevented from establishing a home in certain genomic contexts. For some genes, we expect these influences to be quite strong, but for others, they might be weaker or non-existent. The output from PanForest is two-fold. Firstly, PanForest reports the intrinsic predictability of each gene. Secondly, PanForest reports which other genes are most influential in predicting the presence or absence of the focal gene. This two-fold output enables the user to identify communities of co-occurring genes on the one hand, or genes that avoid being present together in the same genomes, within the entire pangenome. We highlight PanForest’s potential for discovery, by superimposing information about genes’ antimicrobial properties onto the prediction networks inferred by PanForest. Our results show that genes conferring resistance to a particular drug are more likely to either co-occur with, or exclude, other genes for the same drug resistance, compared to their associations with genes lacking this resistance function. As a case study, this shows the potential of this software to identify candidates for AMR genes and suggests that similar candidates can be inferred in other fields.

## 2 Software and implementation

PanForest takes a gene presence–absence matrix, such as those produced by Panaroo (Tonkin-Hill *et al.* 2020) or Roary (Page *et al.* 2015), as input. The program process_matrix.py filters and modifies the matrix in three ways. In the first step, it filters out genes that are present with a frequency that is greater than or less than a user-defined threshold. Secondly, genes and genomes with identical presence–absence patterns are merged into gene family groups and genome groups respectively, both with new unique identifiers. The set of identical genes and genomes that make up each gene family group and genome group are produced as output along with the processed matrix. Lastly, the names of each gene in the matrix are replaced with the number 1 (whilst preserving gene family names) and empty values replaced with 0 to represent absence.

The processed matrix serves as the input to PanForest, which uses the patterns contained within the matrix to make informed predictions of gene occurrences in genomes using the random forest algorithm. Looping through each gene in the portion of the pangenome where gene frequency is both above and below the given thresholds of genomes, the genomes are split randomly into a training set (80%) and a test set (20%) stratified by the gene being predicted. Splitting the data into a training set and test set is essential because the model is trained exclusively on the training set. The test set allows us to ask how well the model performs when applied to data on which it has not been trained, thus how general its properties are to the dataset as a whole. Stratification in this case means that roughly 20% of the genomes with the focal gene present as well as 20% of the genomes where the focal gene is absent will be in the test set. This kind of stratification approach is essential to produce a training and test set that are balanced in terms of their representation of genes and genomes, allowing accurate assessment of model performance. The Random Forest classifier model is generated from the set of predictor genes, taking a sample of size equal to the square root of the total number of predictor genes at each node, according to standard best practices (Pedregosa *et al.* 2011). Trees are built until one of two conditions is reached, depending on user arguments: The first condition is that a maximum depth of the tree is reached. In this case, trees are generated until leaves are 100% pure (contain only genomes where the gene is present or those where they are absent) or the maximum depth is hit. The second condition causes tree construction to halt if a specified minimum purity increase is set by the user. Stopping occurs if no gene in the random sample can create a split that sufficiently reduces the GINI impurity to meet the given threshold. The ‘Maximum Depth’ strategy requires hyperparameter tuning which involves the user testing different maximum depths for the decision trees and selecting the depth that best suits their dataset. For example, users may run their analyses over several sets of parameters and select the results with highest F1 scores. The alternative strategy of supplying a minimum purity decrease is more general as it relies on a pre-defined criterion (improvement in homogeneity) for stopping. Its generality stems from its ability to adapt in a dataset-specific manner. Building decision trees in either way ensures that randomly sampled but highly informative combinations of genes allow the prediction of a focal gene. Users may also supply both a minimum purity decrease and a maximum depth. In this case, whichever condition is satisfied first signals the stopping of the building of the tree.

Furthermore, PanForest supports parallel processing through the generation of trees in parallel according to the number of threads allocated to the program. This feature enhances the program’s usability, particularly for large, complex pangenome datasets.

Once the given number of trees is generated, they collectively form a predictive model of a gene’s presence or absence in a genome. Each genome in the test set is subjected to analysis by the predictive model. Each focal gene has its presence or absence predicted according to the majority vote of all trees in the Random Forest, following the standard practice of a majority vote in Random Forest classifiers (Ho 1995). Model performance is measured by accuracy, recall, precision, and *F*1 score (Van Rijsbergen 1979). Also recorded is the GINI importance (Breiman *et al.* 1984) of each predictor gene in the model for each focal gene. Collectively, these outputs constitute a comprehensive analysis of gene presence predictability and feature analysis. The results are calculated for each gene, and the results are written to a file. This ensures that the results are comprehensive while also remaining accessible to researchers in the field.

Several supplementary programs are included that can process the results in ways that users may find useful. One of the key features of the supplementary tools is their ability to generate graphs from the outputs of the Random Forest analysis. In these graphs, the nodes are gene families, or if merged, the nodes represent patterns of gene family presence and absence. The edges in these graphs signify the predictive relationships between the constituent gene families. This means that if two nodes are connected by an edge, then the presence or absence of one of these nodes is a significant predictor of the presence or absence of the other node. Users can import the networks generated by PanForest into freely available programs including Cytoscape (Shannon *et al.* 2003) and Gephi (Bastian *et al.* 2009). PanForest also allows users to calculate the ‘D statistic’ (Fritz and Purvis 2010) for each gene, approximating how closely correlated the distribution of gene presence/absence is with the phylogeny, which must be user supplied. The tree used in this study was subsampled from Beavan *et al.* (2024) Finally, a script to set up a mySQL [SQLite3 (Hipp 2020)] database is also included so users can probe the results according to SQL queries. By importing PanForest networks into these software packages, users can visually investigate the complex relationships between individual gene families, communities of families and the overall structure of influences within a pangenome. Overall, the supplementary programs enable a more comprehensive and subtle understanding of how pangenomes arise and are maintained and enable a more intuitive presentation of the data.

Though designed to operate on gene presence–absence matrices in relation to pangenomes, this method is, in principle, applicable to any form of discrete data that can be arranged in a matrix. We envisage future analysis incorporating additional data such as environmental conditions, pathogenicity, single nucleotide polymorphism data, promoter regions and physiological features in addition to any other features of genomes that users may consider.

## 3 Materials and methods

### 3.1 Dataset construction

The pangenome of 2241 *Escherichia coli* genomes inferred in Beavan *et al.* (2024) was subsampled according to the maximum likelihood tree they inferred by arbitrarily including one out of the two tips with the smallest distance between them sequentially until only 1000 genomes remained. The gene presence–absence matrix inferred in Beavan *et al.* (2024) was then filtered so that it contained only these 1000 genomes, and any genes present only in the removed strains were also removed. We chose to subsample to 1000 genomes to show what was possible with a dataset of a size that can be easily compared with potential users’ datasets. It also allows each genome to be less closely related, thus retaining the phylogenetic scope of inferred relationships without having to include so many genomes in the study.

### 3.2 PanForest hyperparamaterization

To demonstrate the different ways in which hyperparameters can be specified using PanForest, a titration of ‘minimum purity increase’ was performed and results were compared with those using the parameters used by Beavan *et al.* (2024), of 1000 trees at a maximum of 16 depth. Other than these hyperparameters, all parameters in PanForest were left as default. The number of accurately predicted genes for each set of hyperparameters was then compared, as defined by genes with an *F*1 score greater than or equal to 0.9 and a *D* statistic greater than 0, over 10 repeated analyses.

### 3.3 AMR ontology assignation

To infer the association of gene families with resistance to antimicrobial drugs, a sequence similarity approach was undertaken. The Comprehensive Antibiotic Resistance Database (CARD) ontology version 4.0 was downloaded and a representative sequence for each gene family present in the 1000 genome *E. coli* pangenome, according to the Panaroo-generated ‘pangenome_reference.fa’ file was compared to the set of genes associated with AMR in the CARD database using BlastX (Altschul *et al.* 1990, 1997) with default settings, saving only alignments with an *E*-value less than 10^−10^. Thus, each gene in the *E. coli* pangenome was associated with 0 or more Antibiotic Resistance Ontology (ARO) terms according to the genes to which they are homologous. This was further generalized by parsing the ‘aro.obo’ file from the CARD database to associate each of these gene ARO terms with the ARO term for the drug they confer resistance to. This information was appended to the network files generated from PanForest. In PanForest genes with identical presence–absence patterns across all genomes are collapsed and treated as a single entity. After prediction networks were inferred by PanForest, those sets of genes were all separated into different nodes and assumed to associatively predict each other according to the subsidiary program ‘expand_non_unique.py’.dog.

## 4 Results

### 4.1 PanForest hyperparameterization and runtime

**Figure 1:**
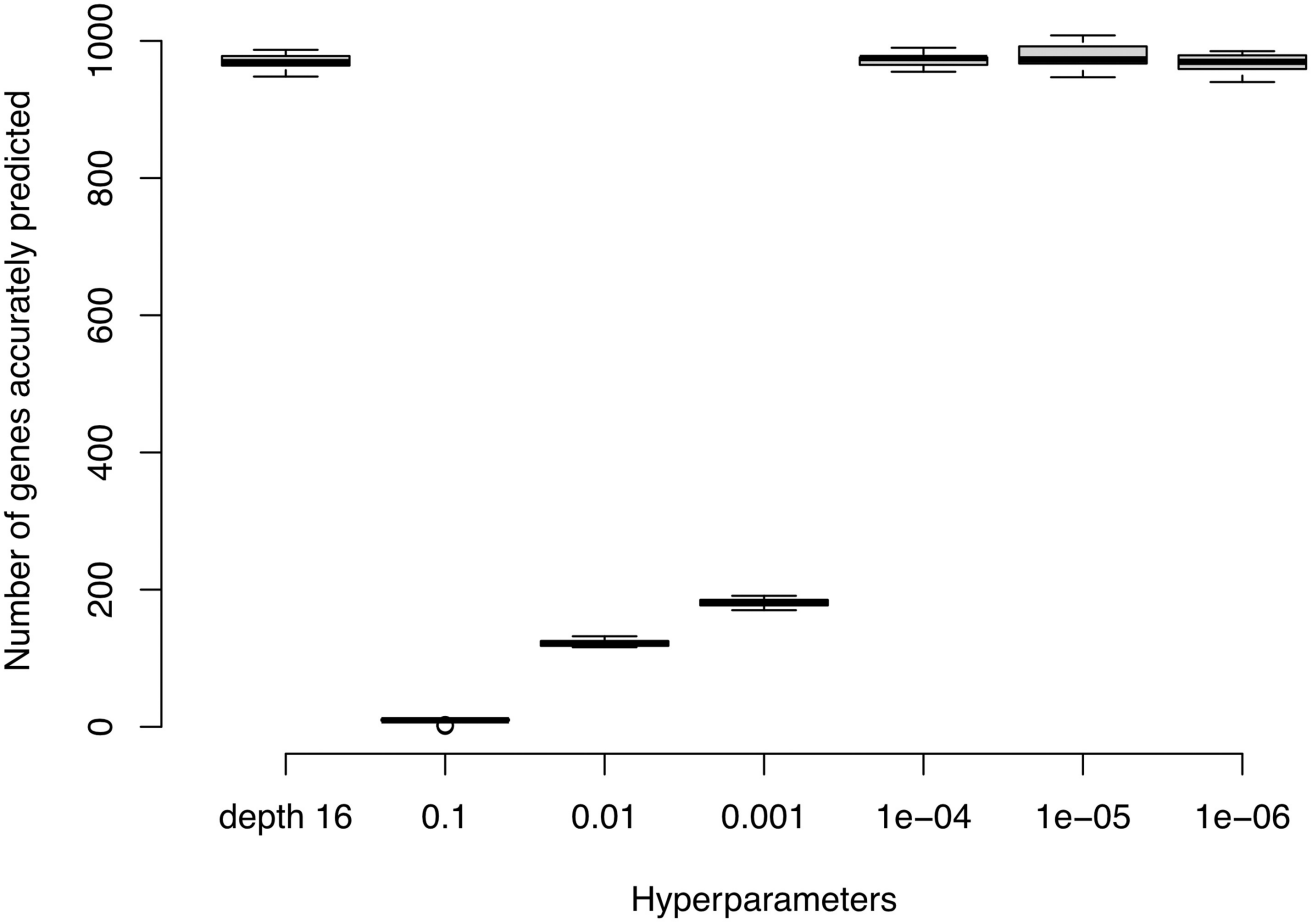
As the minimum purity increase decreases (*X* axis), the performance improves until 10^−4^ at which point it is indistinguishable from trees at a maximum depth of 16, previously determined as optimum in Beavan *et al.* (2024). At minimum purity increase less than this, there is no further improvement in performance but no evidence of overfitting either. Accurately depicted genes are those with an *F*1 statistic greater than or equal to 0.9 and a *D* statistic of at least 0. Each box and whiskers represent 10 repeated analyses with the median at the thick black line, the interquartile range marked by the box and the full range, aside from statistical outliers, represented by the whiskers. Outliers are defined as lying over 1.5 times the interquartile range outside the interquartile range and are marked by a hollow circle.

PanForest provides multiple parameterization strategies to accommodate diverse datasets. These are: minimum purity increase (criterion-based, dataset-adaptive); maximum depth (structural constraint, requires optimization); combined approach (whichever condition is met first). To show the effect of hyperparameter specificity, we titrated over a range of minimum purity increase values and compared with the 1000 trees of 16 maximum depth specified by Beavan *et al.* (2024). The number of genes accurately predicted (out of a theoretical maximum of 10 840 accessory genes with *D* > 0), as defined by the test set *F*1 score >0.9 and a *D* score >0 (which excludes genes whose distribution is highly correlated the underlying strain phylogeny) can be compared across this titration and shows that a value of 10^−4^ represents the point at which decreasing the minimum purity increase has a negligible effect (Fig. 1). We note that for a dataset of 1000 genomes, and for binary data, a minimum purity increase of less than 1/10 000 is impossible, so these final three parameter sets represent trees that have been built until nodes are completely made up of genes being either present or absent. The performance of PanForest at this level is similar to the performance at 1000 trees of 16 maximum depth, and we have not found evidence for overfitting. Combined, this suggests that a generalized approach setting the minimum purity increase to a very low value, meaning trees are built until leaves are completely pure, may be a more time-effective way of obtaining the most accurate results without having to go through extensive hyperparameterization. For exploratory analyses or when computational resources permit, users can perform the same titration we demonstrate (Fig. 1) to identify where performance plateaus for their specific dataset.

**Figure 2:**
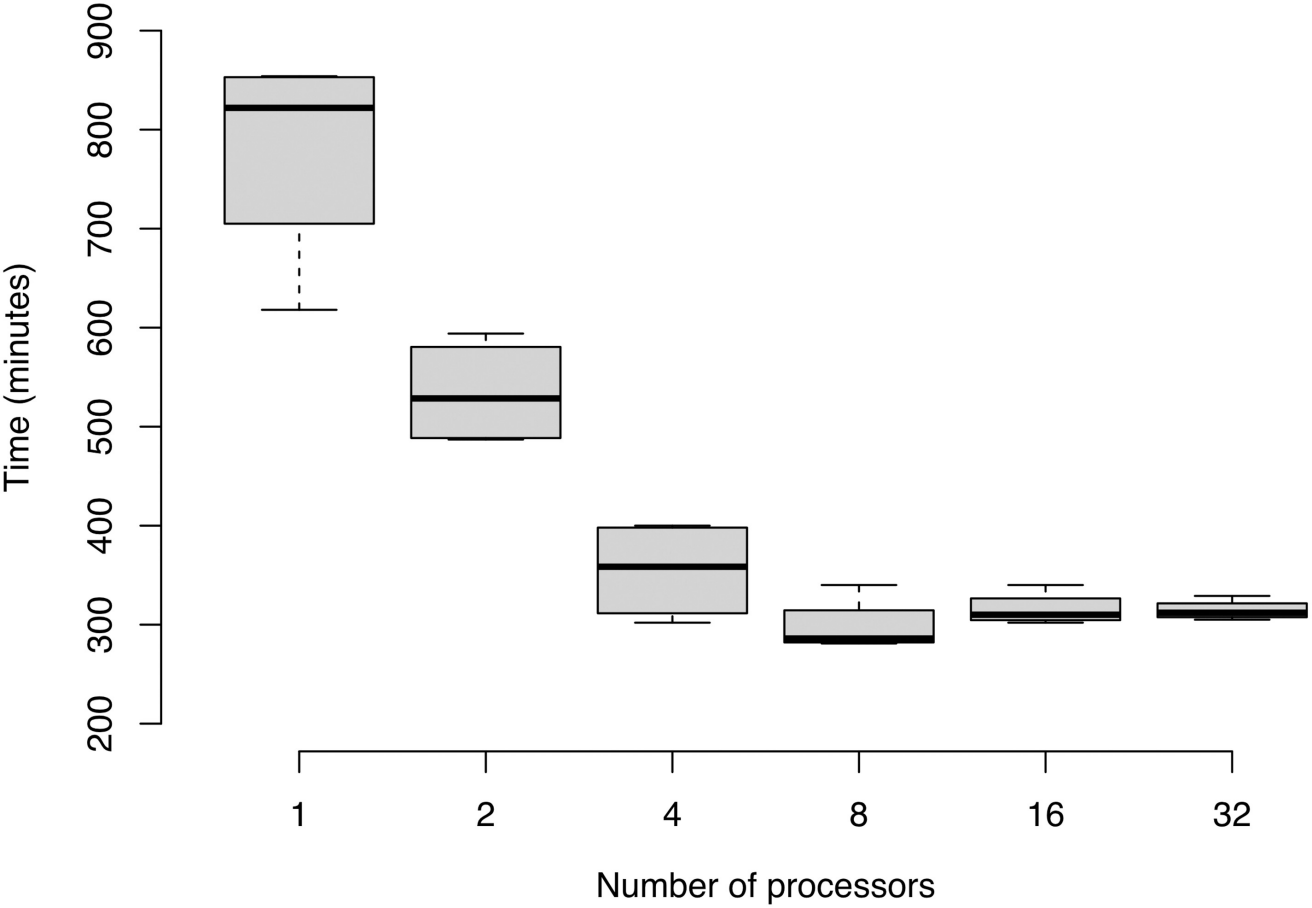
The speed of PanForest increases as more processors are used due to parallel processing. The number of processors is plotted on the *X* axis with the time taken to run the program PanForest.py plotted on the *Y* axis using a logarithmic scale. Each set of box and whiskers represents the time taken for four identical replicated runs per number of processors. The dark line is the median time taken with the outer bounds of the box representing the interquartile range. The whiskers mark the bounds of the range. We would expect analyses to take longer as the size of the pangenome increases because (i) more accessory genes would need to be predicted and (ii) they may require a greater maximum depth of trees for model training.

We evaluated the speed of execution of the PanForest program by titrating over a range from 1 to 32 processors, with three technical replicates of each run (Fig. 2). Analysis times decrease as more processors are used but this benefit diminishes at greater numbers of threads, with no improvement evident above between eight and 16 processors. The median time spent to analyse this dataset using eight processors, was 4 h 46 min.

### 4.2 PanForest outlines the complex predictive relationships of AMR genes

We applied PanForest to the question of how AMR genes form predictive relationships. Gene families present in the predictive gene presence/absence networks inferred by PanForest using a minimum purity increase of 10^−4^ were associated with AMR ontology (ARO) terms for specific drugs according to blast similarity comparisons with the CARD database (Alcock *et al.* 2023). Using PanForest, we tested the hypotheses that (A) genes associated with a specific drug’s ARO term are more likely to be associated with other genes associated with that term than with genes associated with a different ARO term and (B) that those same genes are also more likely to be predictably avoiding other genes associated with that term than genes associated with other terms. The first hypothesis is likely because several genes may be involved in a pathway that only confers resistance when all or several parts of said pathway are present and active. The second may suggest that resistance to a given drug may be conferred in several redundant ways. That is, if a bacterial strain is resistant to a drug via gene A, it does not need to possess gene B, and *vice versa*.

To test these hypotheses, the associative and avoiding predictive relationships identified across 10 repeated analyses were each categorized as either a cis match, if the same ARO term was associated with both genes in the relationship, or a trans match if mutually exclusive sets of ARO terms were associated with each of the two genes. Relationships involving genes with no associated ARO term were excluded from this analysis. A Mann–Whitney *U*-test was performed to compare the median number of cis matches for each ARO term with trans matches for each other term. For associative relationships, we found that a gene was more likely to be predictive or to predict a gene with the same ARO term than one with a different term (*W* = 1 447 644, *P*-value = 4.601e−13). Similarly, genes associated with an ARO term are more likely to avoid genes associated with the same term than they are to avoid genes associated with another term or no terms (*W* = 350 482, *P*-value <2.2e−16). According to the pangenome data and the method of analysis, we reject both null hypotheses of no coincident association with, or no avoidance between genes with the same ARO terms.

**Figure 3:**
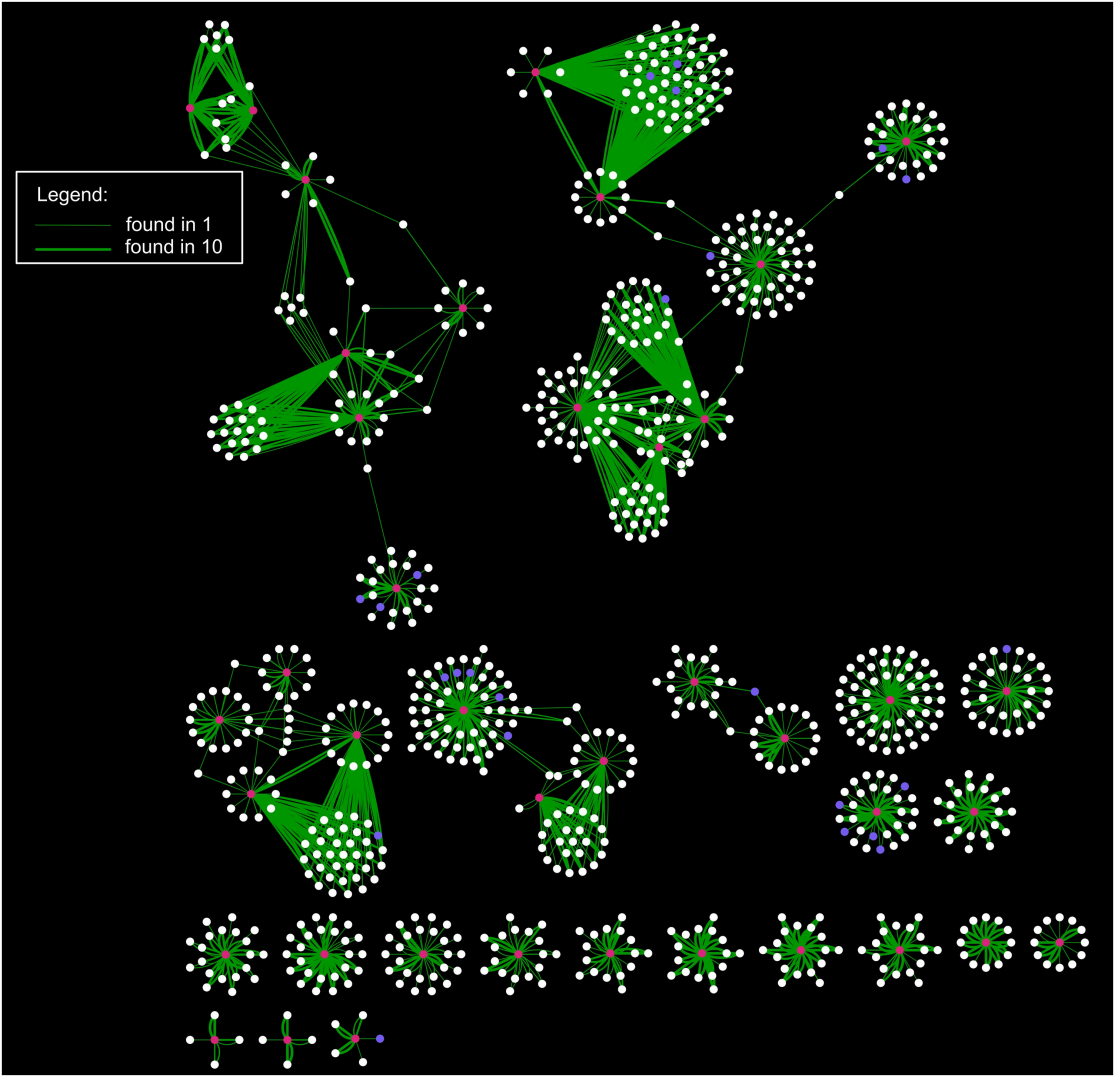
Associative relationships between Metronidazole resistance associated genes (red nodes) and genes are predicted by them, or predict them, show many links with genes not associated with any antibiotic resistance (white nodes), and some genes associated with resistance to other drugs (blue nodes). Edge thickness is proportional to the number of repeated analyses in which a predictive relationship was found between the two connected nodes (see legend). Only the predictive relationships associated with accurately predicted (*F*1 > 0.9, *D* > 0) are plotted.

Given that a gene is more likely to be associated with another gene sharing the same ARO term, we asked what other genes are associated with or avoiding known AMR genes in a specific example. We investigated resistance to Metronidazole (RO:3000689), an antibiotic applied to infections of anaerobically respiring pathogens, which was found to be associated with 153 genes out of the 53 909 genes in this *E. coli* pangenome, and extracted associative predictive relationships from the full set of results inferred across 10 replicate analyses (Fig. 3). The filtered network features many relationships between genes that share the Metronidazole resistance term, such as a connection between the gene family ‘btuD_1∼∼∼irtA∼∼∼btuD_4’ (the tildes here are features of annotation by Panaroo, splitting different ways the gene family might be named according to homology groups) with functions annotated as ‘Vitamin B12 import ATP-binding protein BtuD; hypothetical protein Iron import ATP-binding/permease protein IrtA’ and a gene family labelled ‘group_3859’, annotated as ‘Putative multidrug export ATP-binding/permease protein’, which was found in all ten replicate analyses. However, interestingly, it also shows hundreds of genes not found to be associated with Metronidazole resistance that are predictably found in the same genomes as the Metronidazole resistance-conferring genes. Eight hundred ninety-six gene families’ presence either predicted or were predicted by the presence of the 40 Metronidazole resistance genes found in the *E. coli* pangenome. Of these, many were not found in all the replicated analyses but 210 were reliably found across all 10 repeats. This demonstrates that AMR genes are embedded within much larger, stable networks of genetic associations that extend far beyond the resistance mechanisms themselves.

**Figure 4:**
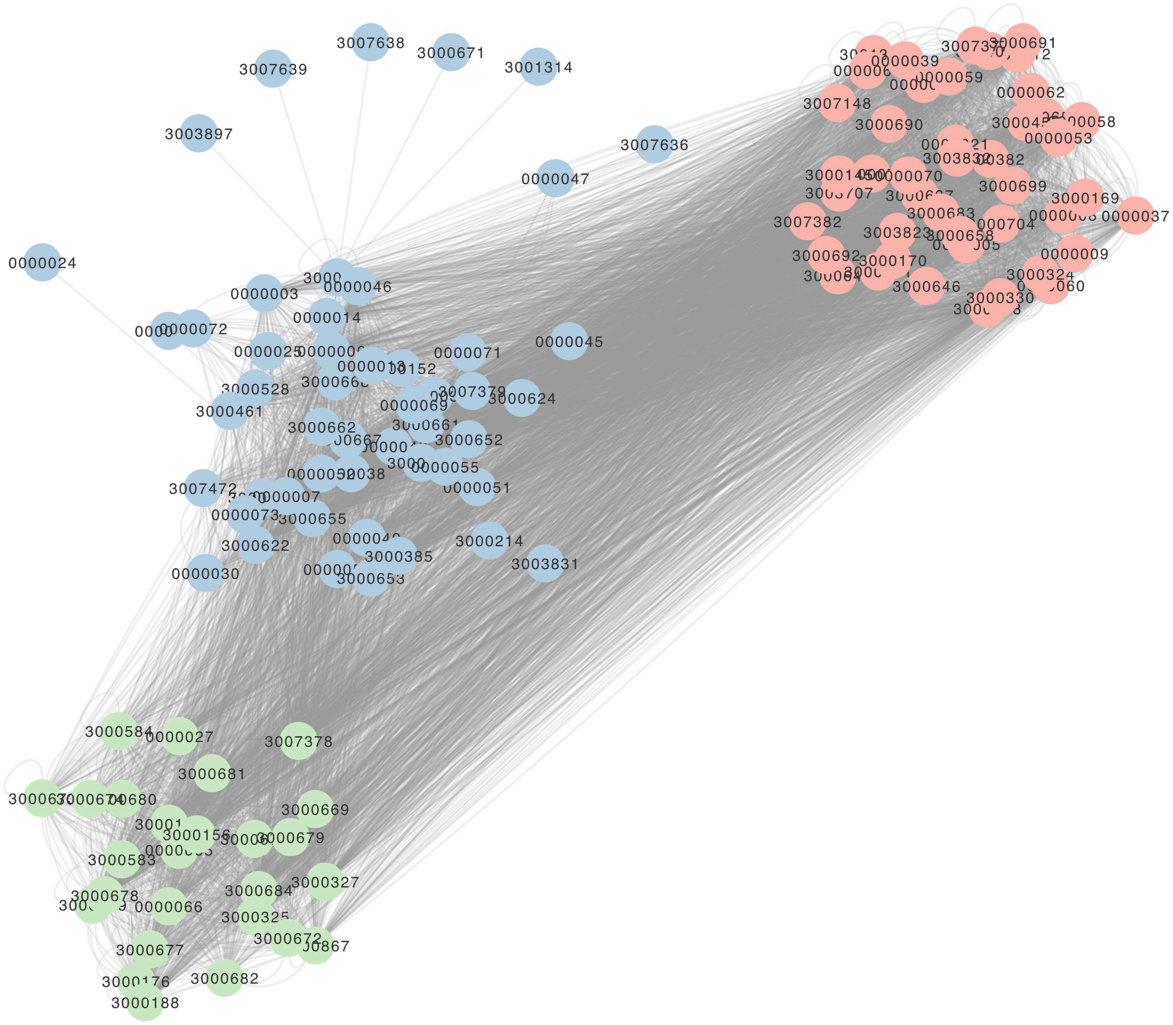
All ARO drug resistance terms found in the *E. coli* pangenome form a single connected component. Each node represents an ARO term according to its label and nodes are coloured according to the community assigned by ‘Glay’ clustering. An edge represents an associative, predictive relationship (*F*1 > 0.9, *D* > 0) found between two genes in at least one of the repeated analyses, given that a different ARO term was associated with each node connected by the edge.

Finally, we used PanForest to highlight the extent to which AMR phenotypes are interconnected. By analysing the associative gene predictive networks produced by PanForest, we counted the number of edges connecting each pair of drug resistance ARO terms and visualized these associations as a network (Fig. 4). All ARO terms from a single connected component within which three clusters were identified by GLay clustering (Su *et al.* 2010). This result reveals the complex, interconnected nature of AMR phenotypes from a pangenome perspective, demonstrating that resistance to different antimicrobials does not arise through discrete, independent events but rather through a web of genetic associations that link resistance mechanisms across multiple drug classes.

## 5 Discussion

In this study, we have described PanForest, a set of tools designed for identification of repeated patterns of gene co-occurrence and avoidance relationships during the evolution of dynamic pangenomes. We demonstrate the computational efficiency of the software for large-scale analyses, as it completed analysis of a pangenome consisting of 1000 *E. coli* genomes in less than 5 h using eight processors. Additionally, we show that setting a very low minimum purity increase threshold provided accurate results—at least in this case—reducing the need for time-consuming optimization of the number of trees and maximum tree depth parameters. Using PanForest requires careful consideration of potential confounding factors, though the software provides tools to address these issues. For example, genes that evolve in a manner consistent with the underlying phylogeny of the organisms studied may seem to be functionally linked, but instead they may have simply been co-inherited through one or a few phylogenetic events. Therefore, we recommend using the phylogenetic *D* statistic to exclude genes whose distribution is highly correlated with the underlying cell phylogeny from predictive analyses (Fritz and Purvis 2010, Whelan *et al.* 2020). A strict cut-off of *D* = 0 has been previously demonstrated to be sufficient for filtering such phylogenetically constrained genes in a larger version of this dataset (Beavan *et al.* 2024). Similarly, based on previous findings, we used an *F*1 score of 0.9 as a threshold above which we define a gene as ‘predictable’. However, PanForest produces a full table of results from which it later filters according to user-defined thresholds of *F*1 and *D* score. As such, it is easy to experiment with different thresholds without having to run more analyses. Results using the same *F*1 and *D* score cut-offs are directly comparable, so should be kept constant across datasets for comparison.

By their nature, Random Forests analyses are stochastic. *F*1 scores and GINI importances will vary across repeated runs. Here, we perform the same analysis, from different, random seeds, 10 times. This is standard practise in many aspects of biology and something we encourage because it can be informative to know which genes most reliably predict a gene of interest. To emphasize this, we found 896 genes to be predictively related to a gene associated with Metronidazole resistance, of which 210 were found in all 10 repeated analyses. We consider these genes the ones with the most substantial evidence of predictive relationships, which is reflected in Fig. 3 by the edge thickness.

It is also important to consider the diversity of the input pangenome. We expect that including a large number of genomes that are closely related (e.g. genomes from a single disease outbreak), would bias results to favour those relationships most often encountered in that clade. Finally, genes in close physical proximity on the chromosome, or genes that share a plasmid are likely to predict each other in an associative manner. Previous findings suggest that a substantial number of associative relationships are between genes that are closely physically linked, but there are many relationships inferred between genes that are not (Beavan *et al.* 2024). Physical linkage should thus be considered by users when evaluating the results of PanForest. When these considerations are properly addressed, PanForest provides a powerful and reliable tool for dissecting the complex genetic networks underlying pangenome evolution across diverse research applications.

Other methods effectively leverage the gene presence/absence variation in pangenomes to identify statistically significant relationships between genes, either as co-occurring or avoiding (Whelan *et al.* 2020, Gavriilidou et al. 2024). Comparing these with PanForest is challenging because there are substantial differences between the two classes of methods (A) those that perform pairwise comparisons of gene presence absence patterns (CoinFinder, GoldFinder), and (B) PanForest, which builds machine-learned multivariate predictive models. The benefit of the second approach is fourfold. First, PanForest captures combinatorial, higher order relationships between genes, using decision trees. Second, *F*1 scores (and other performance metrics produced by PanForest) distinguish how well each gene can be predicted, as opposed to a binary classification of whether two genes share a statistically significant relationship. This allows users to rank genes according to how well they can be predicted. Third, GINI importances can be treated similarly, allowing users to rank genes according to how much they contribute to the accurate prediction of a gene of interest. Fourth, unlike pairwise methods, the Random Forest algorithm allows for predictions to be made in one direction and not the other, which highlights cases of asymmetric relationships between genes in a pangenome. While both sets of methods can produce apparently similar networks of gene–gene associations, the differences noted mean that PanForest allows us to view the data in substantially different ways.

We also demonstrate PanForest’s practical application through analysis of antimicrobial resistance gene networks. While this analysis serves primarily as a methodological demonstration rather than a comprehensive search for resistance candidates, the results reveal significant biological insights. The identification of 210 gene families consistently associated with Metronidazole resistance genes across all 10 analytical replicates exemplifies the tool’s capacity to uncover robust genetic associations. As we have previously argued (Beavan *et al.* 2024), such reproducible patterns of gene co-occurrence suggest deterministic evolutionary processes that extend far beyond random chance events. This approach, demonstrated here with Metronidazole, can be readily applied to any AMR phenotype, offering researchers a systematic framework for mapping the complex genetic networks that underlie microbial adaptation (see Supplementary Files, available as supplementary data at *Bioinformatics* online). PanForest thus provides not only computational efficiency for large-scale pangenomic analyses, but also the analytical depth necessary to reveal the evolutionary forces shaping microbial genomes.

## Supplementary Material

btag005_Supplementary_Data

## Data Availability

The software if freely available with a full manual and can be found with at www.github.com/alanbeavan/PanForest DOI: https://doi.org/10.5281/zenodo.17865482. It requires python3.9+ and R version 4.2+ with some Python modules and R libraries indicated in the installation instructions in the manual. The combined 10-run networks for both association and avoidance networks with ARO terms added are available as supplementary information.
